# Cardiovascular Endocrinology: Evolving Concepts and Updated Epidemiology of Relevant Diseases

**DOI:** 10.3389/fendo.2021.772876

**Published:** 2021-10-05

**Authors:** Fahimeh Varzideh, Urna Kansakar, Stanislovas S. Jankauskas, Jessica Gambardella, Gaetano Santulli

**Affiliations:** ^1^ Department of Medicine, Division of Cardiology, Wilf Family Cardiovascular Research Institute, Einstein Institute for Aging Research, Albert Einstein College of Medicine, New York, NY, United States; ^2^ Department of Molecular Pharmacology, Einstein-Mount Sinai Diabetes Research Center (ES-DRC), Fleischer Institute for Diabetes and Metabolism (FIDAM), Einstein Institute for Neuroimmunology and Inflammation, Albert Einstein College of Medicine, New York, NY, United States; ^3^ Department of Advanced Biomedical Science, “Federico II” University, and International Translational Research and Medical Education (ITME) Consortium, Naples, Italy

**Keywords:** cardiometabolism, diabetes mellitus, endocrine hypertension, HFpEF, insulin resistance, metabolic syndrome, NAFLD, uremic cardiomyopathy

## What Is Cardiovascular Endocrinology?

We created the new *Cardiovascular Endocrinology* section of *Frontiers in Endocrinology* as the perfect home for high-quality research across all aspects of cardiovascular endocrinology. This interdisciplinary forum will solicit significant advances in areas including, but not limited to:

- Cross-talk between the cardiovascular system and other organs and systems, such as kidney, lungs, thyroid, bone, skeletal muscle, gastrointestinal system, and nervous system (1);
- Involvement of the heart in systemic disorders;
- Autonomic regulation of the heart and the cardiovascular system;
- Stem cell mediated cardiovascular protection;
- Functional roles of hormones, regulatory peptides, and non-coding RNAs produced by (or actin on) the cardiovascular system (2–6).

To give to our Readers an idea of the burden of cardiovascular endocrinology in the clinical scenario, in this inaugural Editorial, we briefly discuss the current epidemiology of some disorders that perfectly fit within the definition of cardiovascular endocrinology (and therefore within the scope of this new Journal): endocrine hypertension, heart failure with preserved ejection fraction (HFpEF), metabolic syndrome, non-alcoholic fatty liver disease (NAFLD), and uremic cardiomyopathy.

## Endocrine Hypertension

Based on conservative estimates, secondary hypertension affects 5-10% of the general hypertensive population, but could be more common than currently recognized (7). More than 50% of children and ~30% of young adults (<40-year-old) who present with hypertension have a secondary cause, and such secondary causes are broadly categorized into renal and endocrine causes (8). We briefly report below the main endocrine causes of endocrine hypertension.

Adrenal-dependent causes include primary aldosteronism [which prevalence has been underestimated for decades and has been recently shown to be ~22% in patients with resistant hypertension (9)], Cushing syndrome (~10-15 per million are affected by the endogenous form, whereas iatrogenic forms are way more common and largely underestimated), Apparent mineralocorticoid excess (AME)/11β-hydroxysteroid dehydrogenase deficiency (an extremely rare disease, also known as Ulick Syndrome, with fewer than 100 cases reported hitherto, resulting from an impaired activity of the enzyme HSD11B2, which normally inactivates cortisol), pheochromocytoma and sympathetic paraganglioma (prevalence: 20-60 per 10.000 patients who present with hypertension) (10).

Parathyroid-dependent causes are essentially represented by hyperparathyroidism (incidence: 4.03 per 10.000 in females and 1.37 in males). Pituitary-dependent causes include Cushing disease due to excessive ACTH secretion and acromegaly (prevalence: 50-70 per million). Other mechanisms leading to endocrine hypertension include secondary hyperaldosteronism (for instance due to renovascular hypertension) and thyroid-dependent causes (both hypothyroidism and hyperthyroidism) (11).

## HFpEF

Several studies estimate that as many as 40-60% of patients with heart failure (HF) have a normal (≥50%) LVEF (12). The proportion of patients with HF who have HFpEF is higher in older adults and appears to be increasing by about 1% annually relative to that of HF with reduced ejection fraction (HFrEF) (13). Most patients with HFpEF display normal left ventricular volumes and evidence of diastolic dysfunction, such as elevated filling pressures at rest or with exertion.

The pathophysiological understanding of HFpEF is still limited. Recent reports have shown that many HFpEF patients exhibit signs of non-resolving inflammation, endothelial dysfunction, insulin resistance, hyperlipidemia, and multiorgan defects (14). At a cellular level, cardiomyocytes in patients with HFpEF are thicker and shorter than normal cells, collagen content is increased, and recent histologic evaluations have revealed reductions in myocardial capillary density alongside lymphatic dysfunction (15). Furthermore, substantial evidence indicates that obesity-related HFpEF may result from increased mineralocorticoid signaling, adipokines imbalance, and neprilysin overactivity (16).

## Metabolic Syndrome

There are several definitions for metabolic syndrome; the one elaborated by the National Cholesterol Education Program (NCEP) Adult Treatment Panel III (ATPIII) is one of the most widely used, confirming the diagnosis in presence of any three of the following five traits:

-Abdominal obesity, defined as a waist circumference ≥102 cm (40 in) in men and ≥88 cm (35 in) in females;
-Blood pressure ≥130/85 mmHg or drug treatment for hypertension;
-Fasting plasma glucose (FPG) ≥100 mg/dL (5.6 mmol/L) or drug treatment for hyperglycemia or diabetes;
-Serum triglycerides ≥150 mg/dL (1.7 mmol/L) or drug treatment for hypertriglyceridemia;
-Serum high-density lipoprotein (HDL) cholesterol <40 mg/dL (1 mmol/L) in males and <50 mg/dL (1.3 mmol/L) in females or drug treatment for low HDL cholesterol.

Metabolic syndrome has become increasingly prevalent in the last years. According to data from the National Health and Nutrition Examination Survey (NHANES) 2011 to 2016 database, 34.7 percent of participants met the above criteria compared with 22 percent in NHANES III (1988 to 1994); prevalence was 19.5% among those aged 20 to 39 years and increased to 48.6% among those aged at least 60 years (17).

## NAFLD

NAFLD refers to the presence of hepatic steatosis when no other causes for secondary hepatic fat accumulation are present. It represents the most common liver disorder in industrialized countries, with a prevalence of 10 to 46% in the United States, and 6 to 35% (median 20%) worldwide. The diagnosis of NAFLD requires evidence (by imaging or histology) of hepatic steatosis and the exclusion of secondary causes of hepatic fat accumulation, including steatogenic medication (*e.g.* corticosteroids, methotrexate, amiodarone), viral infections (*e.g.* hepatitis C), or hereditary disorders (*e.g.* alpha-1 antitrypsin deficiency, Wilson’s disease); moreover, daily alcohol consumption must not exceed 30g for men and 20g for women.

While metabolic syndrome is a known risk factor for cardiovascular disease and is common in NAFLD patients, NAFLD itself may be independently associated with cardiovascular disease (18). The underlying mechanisms linking NAFLD to cardiovascular disease are very complex and involve a number of different pathways, including insulin resistance, endothelial dysfunction, fibrosis, and alterations in gut microbiota (19).

## Uremic Cardiomyopathy

The burden of cardiovascular disease in patients with end-stage renal disease is significant, with mortality from cardiovascular disease 15 to 30 times higher than the general population. Uremic cardiomyopathy is classically characterized by diastolic dysfunction in association with myocardial fibrosis and left ventricular hypertrophy in patients with chronic kidney disease.

The prevalence of HF in patients with chronic kidney disease populations increases with age, is markedly more common in dialysis patients (prevalence: 31-36%) than in those with normal kidney function (prevalence: 1.8-4.4%), and is inversely proportional to the estimated glomerular filtration rate.

Uremic cardiomyopathy may manifest as a result of hemodynamic overload (both pressure and volume), and a systemic uremic state. Alterations in mineral metabolism, coronary microvascular dysfunction, and the accumulation of substances such as endothelin, parathyroid hormone, tumor necrosis factor alpha, interleukin-1α and interleukin-6, endogenous cardiotonic steroids as cardenolides (oubain and digoxin) and bufadienolides (marinobufagenin and proscillaridin A) contribute to the pathogenesis of uremic cardiomyopathy (20).

## Author Contributions

GS conceived the paper structure, searched for information, and wrote the paper. FV, UK, SSJ, and JG searched for information and wrote the paper. All authors contributed to the article and approved the submitted version.

## Funding

The Santulli’s Lab is supported in part by the National Institutes of Health (NIH: R01-DK123259, R01-HL146691, R01-DK033823, R01-HL159062, R56-AG066431, T32-HL144456, and R00-DK107895, to GS), by the Irma T. Hirschl and Monique Weill-Caulier Trusts (to GS) and by the American Heart Association (AHA-21POST836407 to SSJ and AHA-20POST35211151 to JG).

## Conflict of Interest

The authors declare that the research was conducted in the absence of any commercial or financial relationships that could be construed as a potential conflict of interest.

## Publisher’s Note

All claims expressed in this article are solely those of the authors and do not necessarily represent those of their affiliated organizations, or those of the publisher, the editors and the reviewers. Any product that may be evaluated in this article, or claim that may be made by its manufacturer, is not guaranteed or endorsed by the publisher.
